# The Therapeutic Effect of Negative Pressure in Treating Femoral Head Necrosis in Rabbits

**DOI:** 10.1371/journal.pone.0055745

**Published:** 2013-01-31

**Authors:** Yin-gang Zhang, Xuezhi Wang, Zhi Yang, Hong Zhang, Miao Liu, Yushen Qiu, Xiong Guo

**Affiliations:** 1 Department of Orthopedics, First Affiliated Hospital of Medical College of Xi'an Jiaotong University, Xi'an, China; 2 Department of General Surgery, Zhenghe Hospital, Xi'an, China; 3 Key Laboratory of Environment and Genes Related to Diseases of Ministry of Education, Xi'an Jiaotong University, Xi'an, China; Universidad Europea de Madrid, Spain

## Abstract

Because negative pressure can stimulate vascular proliferation, improve blood circulation and promote osteogenic differentiation of bone marrow stromal cells, we investigated the therapeutic effect of negative pressure on femoral head necrosis (FHN) in a rabbit model. Animals were divided into four groups (n = 60/group): [1] model control, [2] core decompression, [3] negative pressure and [4] normal control groups. Histological investigation revealed that at 4 and 8 weeks postoperatively, improvements were observed in trabecular bone shape, empty lacunae and numbers of bone marrow hematopoietic cells and fat cells in the negative pressure group compared to the core decompression group. At week 8, there were no significant differences between the negative pressure and normal control groups. Immunohistochemistry staining revealed higher expression of vascular endothelial growth factor (VEGF) and bone morphogenetic protein-2 (BMP-2) in the femoral heads in the negative pressure group compared with the core decompression group. Transmission electron microscopy revealed that cell organelles were further developed in the negative pressure group compared with the core decompression group. Microvascular ink staining revealed an increased number of bone marrow ink-stained blood vessels, a thicker vascular lumen and increased microvascular density in the negative pressure group relative to the core decompression group. Real-time polymerase chain reaction revealed that expression levels of both VEGF and BMP-2 were higher in the negative pressure group compared with the core decompression group. In summary, negative pressure has a therapeutic effect on FHN. This effect is superior to core decompression, indicating that negative pressure is a potentially valuable method for treating early FHN.

## Introduction

Organisms are continuously exposed to external mechanical stimuli, and within the body are required to maintain a number of static or dynamic mechanical interactions. The effect of external physical force and internal stress on cell growth, morphogenesis and differentiation has attracted much scientific attention. In a simple example, physical exercise causes the skeletal muscle cell volume to increase, becoming hypertrophic. Similarly, in patients with hypertension, elevated blood pressure resulting from mechanical stimulation, causes vascular smooth muscle cell and cardiac myocyte hypertrophy. In another example, the development, functional maintenance and remodeling of cartilage tissue and tendon requires mechanical stimulation. With continued in-depth research, mechanical stress has been found to regulate many physiological and pathological processes. In fact, cell mechanical stimuli can regulate many functions, including growth, differentiation, gene expression, protein synthesis and apoptosis.

**Figure 1:**
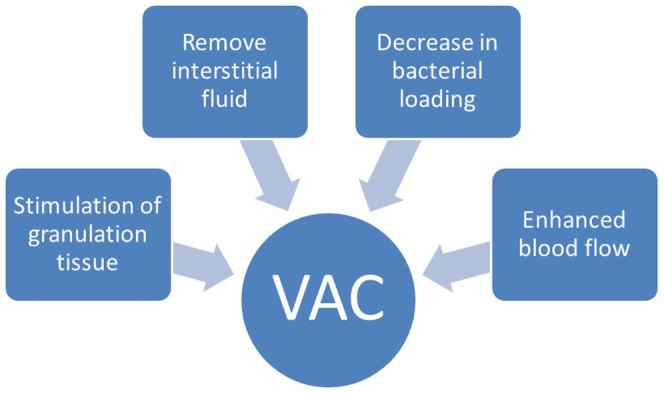
Diagrammatic illustration of the biological effects of VAC.

During the exploration of disease treatments, many techniques have been investigated, including vacuum-assisted closure (VAC), which was introduced in the 1950s. Following improvements in the late 1980s, the technique was used to treat chronic, complex wounds and its significant therapeutic effect has drawn increased attention. The range of diseases for which VAC treatment is suited is continuously expanding. VAC currently plays an important role in the treatment of limb trauma, soft tissue defects, chronic osteomyelitis, compartment syndrome and limb replantation [1]. The main working principle of the VAC technique is shown in Figure 1. By exerting mechanical and subsequent biological effects on soft tissues, negative pressure can stimulate angiogenesis, improve blood circulation, promote growth of both cells and granulation tissue, and accelerate the healing of tissue wounds [2].

Hemodynamic factors and stress stimuli are essential during the processes of bone tissue regeneration and reconstruction, and applying negative pressure can mediate soft tissue repair via these factors. From a cell biology perspective, previous studies have investigated the effect of applying intermittent negative pressure on the proliferation and differentiation of bone marrow stromal cells (BMSCs) and on their expression levels of osteoblast-related genes. These studies found that intermittent negative pressure promotes osteogenic differentiation, up-regulates expression of osteoblast-related genes and enhances the osteogenic activity of BMSCs, thereby supporting the cellular influence of negative pressure on the repair of bone tissue [3]–[5].

Femoral head necrosis (FNH) is a devastating degenerative disease that occurs primarily in young and middle-aged individuals. In the later stages, the disease can lead to femoral head collapse and secondary degenerative arthritis, thereby seriously affecting the patient's quality of life. FHN has an extremely high disability rate, and most patients eventually require total hip arthroplasty. Currently, the use of corticosteroids is the primary causative factor for FHN [6]. A large number of animal experiments and clinical studies [7] have demonstrated that possible pathogenic mechanisms include fat metabolic disorders and fat embolism, vasculitis and microvascular injury, a pre-coagulation condition and intravascular coagulation, high intraosseous pressure and venous stasis, bone cell degeneration and necrosis, and cytotoxicity-induced osteoporosis. Overall, the pathogenesis of FHN is extremely complex and is probably the result of a combination of multiple factors. Given that negative pressure can stimulate angiogenesis, improve blood circulation and promote osteogenic differentiation of BMSCs, we theorized that application of this technique might exert a therapeutic effect on FHN. With steroid-induced FHN, the local supply of blood to the femoral head is interrupted, and the bone undergoes necrosis followed by a repair reaction within the femoral head. First, the junction between the dead bone edge and the surrounding living tissue begins to repair itself via the process of angiogenesis, dead bone resorption and new bone formation. Angiogenic and osteogenic factors, particularly vascular endothelial growth factor (VEGF) and bone morphogenetic protein-2 (BMP-2), play an important role in femoral head repair. Both VEGF and BMP-2 have relatively strong repair capacities in their respective families [8]–[10]. In the present study, we established an animal model of early FHN in rabbits. We evaluated the therapeutic effect of negative pressure on FHN using histomorphology and by measuring the dynamics of VEGF and BMP-2 expression.

## Materials and Methods

### Animal model of femoral head ischemic necrosis

This study was performed in strict accordance with the recommendations in the Guide for the Care and Use of Laboratory Animals of the National Institutes of Health, and every effort was made to minimize suffering. The protocol was approved by the Animal Care and Use Committee of Xi'an Jiaotong University. Group size was determined subjectively based on pilot studies, as the study was designed to be descriptive (vs. quantitative) in nature. Three hundred healthy adult male New Zealand White rabbits (3.0–4.0 kg) were provided by the animal center of Xi'an Jiaotong University. Based on a protocol developed by Yamamoto *et al* [11], we optimize the amount and time of endotoxin and methylprednisolone exposure. Each animal received an ear-vein injection of *Escherichia coli* endotoxin (0111: B4; Sigma) at a dose of 10 µg/kg. The endotoxin administration was repeated 24 h later, followed immediately by a gluteal injection of methylprednisolone (US Fama International, Belgium plant, imported drugs registration number H20040338) at a dose of 40 mg/kg for a total of three injections at 24 h intervals. Six weeks after endotoxin administration, each surviving animal underwent a bilateral hip joint MRI scan. Animals with fat deposits in the bilateral bone marrow cavities of the femoral head, with high T1WI, medium T2WI and low T1W2/SPIR signals (T2WI fat signal suppression), were included in the treatment group. Animals with normal bilateral femoral head bone marrow cavities or a unilateral abnormality were excluded from the study. Two rabbits from each treatment group were selected at random and were euthanized by an intravenous injection of air. Routine histological examination of the bilateral femoral heads revealed sparse and thinning trabecular bone and a significant increase in the number of empty lacunae, confirming our success in establishing an animal model to test subsequent treatments.

### Experimental groups

**Figure 2:**
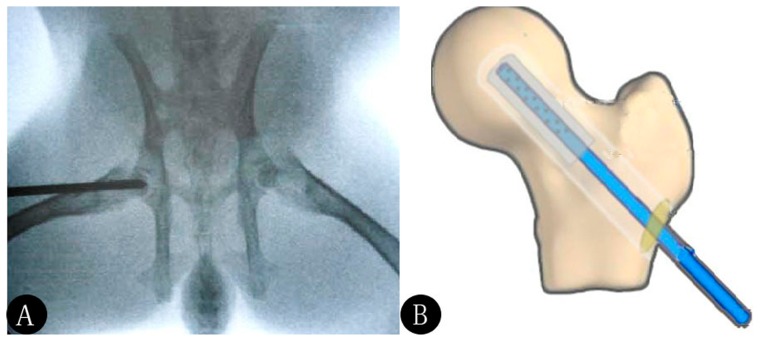
Core decompression surgery. An X-ray scan ensured the depth of decompression (A). A negative pressure connection tube was employed as shown in (B).

A total of 21 animals died within 6 weeks of initial administration of steroids, thereby yielding a mortality rate of 7.0%. The remaining animals were screened by MRI and 180 were selected for subsequent experimentation. These 180 animals were divided randomly into the following three groups (n = 60/group): [1] model control, [2] core decompression and [3] negative pressure groups. In addition, 60 untreated animals were used in a normal control group. Animals were placed in the prone position and were anesthetized with an intramuscular injection of a 1∶1 mixture of ketamine and sumianxin. The skin was incised along the greater trochanter, the muscle and periosteum were separated, and the greater trochanter of the femoral head was drilled using a 2 mm drill, without crossing the boundary surface of the femoral head cartilage. Pathological specimens were removed during drilling. The bone marrow cavity was then completely cleaned. The incision was closed directly in the core decompression group. A custom-made negative pressure connection tube was placed in the animals in the negative pressure group (Figure 2). After surgery, the animals received a daily intramuscular injection of 100,000 units of penicillin for one week to prevent infection. In the negative pressure group, negative pressure was applied after the animals had recovered from the anesthesia. During negative pressure application, the suction tube was connected to a negative pressure instrument to apply suction at 50 kPa for 30 min twice daily. The suction tube was removed six weeks later. Survival rate, mental state, appetite, excretions, weight change and surface infection rate were observed daily in the experimental animals. Experimental animals were weighed weekly and spent two hours each day outside of their cage to increase the scope and level of their activities.

### Histopathology

Four animals were selected randomly from each group, postoperatively at weeks 4 and 8 and were euthanized by an anesthesia overdose. Two animals were subsequently selected at random and bilateral femoral head specimens were obtained for hematoxylin and eosin (H&E) staining and immunohistochemistry. The remaining two animals were processed for transmission electron microscopy (TEM) examination. After H&E staining, bone tissue, bone marrow and any changes in the trabecular bone and fat tissue in the bone marrow were visualized under a light microscope. Using 100× magnification, 10 high-power fields were selected randomly and 50 lacunae were counted in each field. The number of empty lacunae were counted to calculate the percentage of empty lacunae. The diagnostic criteria for osteonecrosis included, diffuse empty lacunae in the trabecular bone or condensed nuclei in the bone cells, combined with necrosis of the surrounding bone marrow cells. Peroxidase-labeled streptavidin (streptavidin-peroxidase, SP) was used for immunohistochemical staining. In addition, BMP-2 and VEGF monoclonal rabbit antibodies (1∶100 dilution with phosphate buffered saline, PBS) and a biotinylated goat anti-rabbit IgG secondary antibody were used. The criterion for a positive result was the presence of yellow-brown reaction product in the cell membrane, cytoplasm, or matrix. As a negative control for staining, PBS solution was used in place of primary antibody, while the remaining steps were identical. For TEM examination, a 1 mm^3^ bone fragment was cut from the subchondral bone area and pre-fixed in glutaraldehyde solution followed by decalcification in 5% EDTA and post-fixation in osmium tetroxide. Semi-thin sections were sliced, stained with uranyl acetate and lead citrate and observed under a PHILIPS CM-120 TE microscope (Philips, The Netherlands).

### Femoral head microvascular features

Eight rabbits were selected randomly from each group, postoperatively at weeks 4 and 8. Animals were anesthetized via an intravenous injection of 2.5% pentobarbital. The lower abdominal aorta was exposed and a tube was inserted. The inferior vena cava (IVC) was then cut and flushed with a heparin-saline solution until clear liquid flowed from the IVC. Gelatin ink (5 g gelatin, 50 ml ink and 50 ml saline solution) was then continuously and slowly infused until the ink returned from the IVC. After ligation of the IVC, animals were euthanized and the left femoral head was removed, fixed in 10% formalin solution and decalcified in 5% EDTA. Samples were paraffin-embedded and 10 µm thickness sections were cut. Under 100× magnification using a microscope, 10 fields were randomly selected in the subchondral compact bone and cancellous bone areas on each sample. Ink-dyed vessels were counted and the average was used to represent microvessel density. Each apparently isolated vascular endothelial cell or endothelial cell cluster approaching the microvascular, was considered as independent microvascular.

### Real-time PCR (RT-PCR)

Six animals from each group were euthanized at weeks 1, 2, 3, 4, 5, 6, 7 and 8, and the femoral heads were removed. Femoral heads were then sawed open along the coronal plane, placed into cryovials and stored in liquid nitrogen. A homogenate of 100 mg of femoral head bone tissue was used for analysis. Mixed tissue was flash-frozen in liquid nitrogen and subsequently ground with a mortar and pestle. Liquid nitrogen was allowed to evaporate, total RNA was extracted using Trizol reagent and the concentration and purity of the resulting RNA was measured using an ultraviolet spectrophotometer. A 2 µg sample of total RNA was used to synthesize cDNA using a reverse transcriptase kit and was added to a quantitative RT-PCR amplification system. The following amplification conditions were used: denaturing at 94°C for 30 s, annealing at 53°C for 30 s and extension at 72°C for 30 s for a total of 50 amplification cycles. Ct values were calculated using a quantitative RT-PCR analysis program. The ΔΔCt value was equal to the Ct objective gene (Ct β-actin) and the relative value of the objective gene was equal to the 2-ΔΔC value. VEGF primer sequences were (forward) 5′-GGAGTACCCTGATGAGATCGA-3′ and (reverse) 5′-CTTTGGTCTGCATTCACATTTGT-3′. BMP-2 primer sequences were (forward) 5′-CGTGAGGATTAGCAGGTCTTTG-3′ and (reverse) 5′-TTTCGCTTGACGCTTTTCTC-3′. β-actin primer sequences were (forward) 5′-AGGCACCAGGG CGTGAT-3′ and (reverse) 5′-TCGTCCCAGTTGGTGACGAT-3′.

### Statistical analysis

Femoral head microvascular density and RT-PCR data are expressed as mean ± standard deviation (SD). Data were analyzed using the SPSS 11.5 statistical software package. An analysis of multivariate variance was used. The Student-Newman-Keuls test was used for pairwise comparisons. Differences with *P*<0.05 are considered statistically significant.

## Results

Seven rabbits died in the latter part of the experiment, including four, two and one from the model control, core decompression and negative pressure groups respectively. No rabbits died in the normal control group.

### Histology

**Figure 3:**
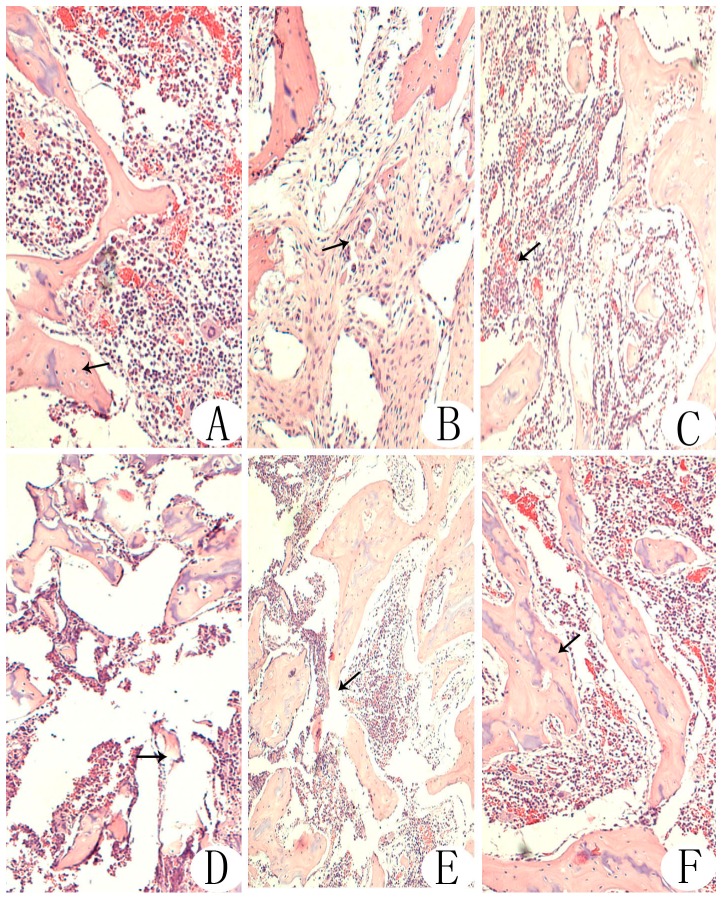
H&E staining of samples from each experimental group. Normal control group (A): Intact, dense, full trabecular bone, clearly visible bone cells in the trabecular bone (arrow), isolated and scattered empty lacunae, abundant bone marrow hematopoietic cells, relatively few fat cells and normal morphology. Model control group (D): Fractured trabecular bone with fragments, an absence of bone cells from some bone lacunae (→), an increased number of empty lacunae, disorganized or absent bone marrow structure and necrosis of a large number of bone marrow cells. Core decompression group, week 4 (B): New capillaries and a small number of ossification centers around a tunnel (→), swelling of the bone marrow compared with the normal control group, an increased number of fat cells, thinning and fractured trabecular bone, a slight increase in the number of empty lacunae and nuclear condensation of a small number of cells. Core decompression group, week 8 (C): An increased number of bone cells in the trabecular bone, an increased number of bone marrow hematopoietic cells (→) and a decreased number of empty lacunae. Negative pressure group, week 4 (E): Disarranged new trabecular bone and a large number of surrounding osteoblasts (→); trabecular bone fracture and necrotic bone marrow cells remain. Negative pressure group, week 8 (F): Intact trabecular bone, clearly visible bone cells in the trabecular bone (→), abundant bone marrow hematopoietic cells, relatively few fat cells and normal morphology resembling normal bone tissue.

**Figure 4:**
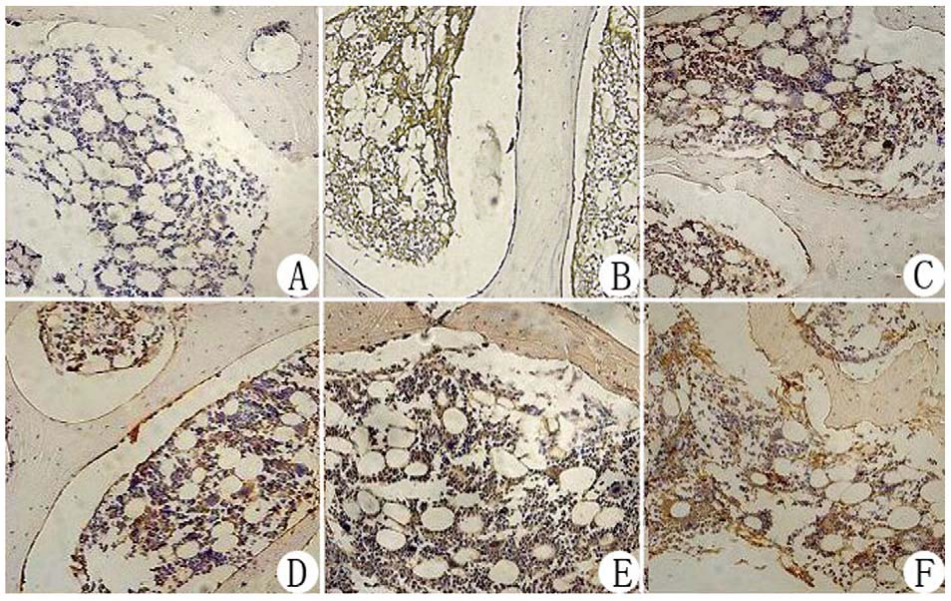
Immunohistochemical staining of VEGF in the femoral heads from each experimental group (200× magnification). Model control group: Low VEGF expression in osteoblasts and vascular endothelial cells in the subchondral bone tissue of the femoral head (A). Normal control group: Some VEGF expression in osteoblasts and vascular endothelial cells in the femoral head (D). Core decompression group, week 4 and week 8: Strong VEGF expression in osteoblasts and vascular endothelial cells in the fibrous granulation tissue (B, C). Negative pressure group: Strong expression at both week 4 and week 8 (E, F), which was higher compared with expression in the core decompression group.

**Figure 5:**
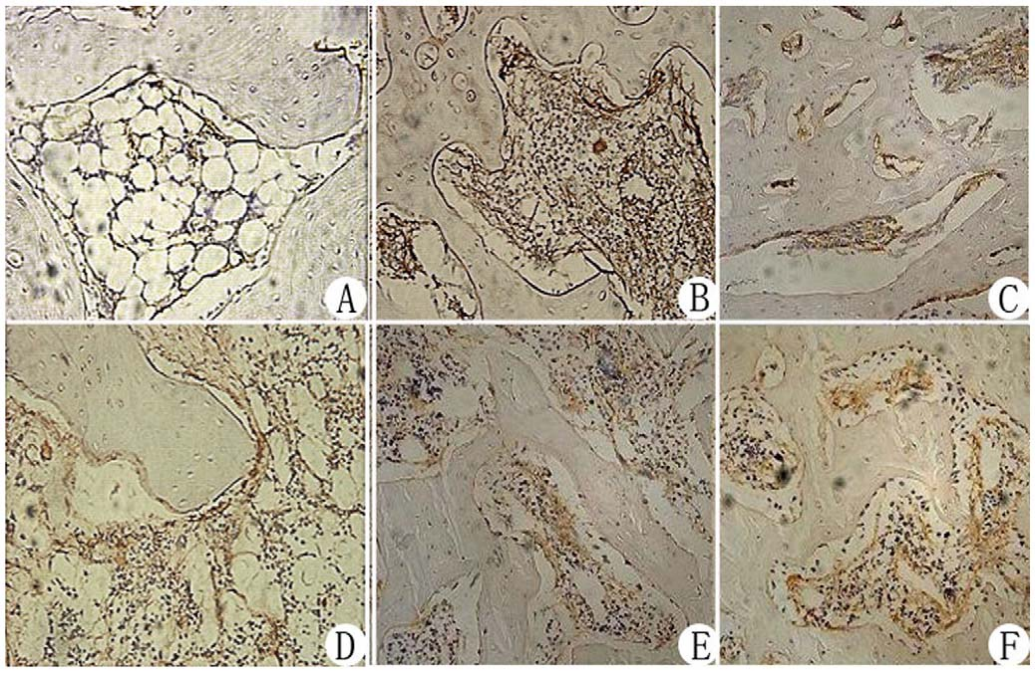
Immunohistochemical staining of BMP-2 in the femoral heads from each experimental group (200× magnification). Model control group: Low BMP-2 expression in osteoblasts and fibroblasts in the subchondral bone tissue of the femoral head (A). Normal control group: BMP-2 expression in the osteoblasts and fibroblasts of the femoral head (D). Core decompression group, week 4 and week 8: Strong BMP-2 expression in osteoblasts and fibroblasts (B, C). Negative pressure group: Strong expression at both week 4 and week 8, which is higher compared with expression in the core decompression group.

**Figure 6:**
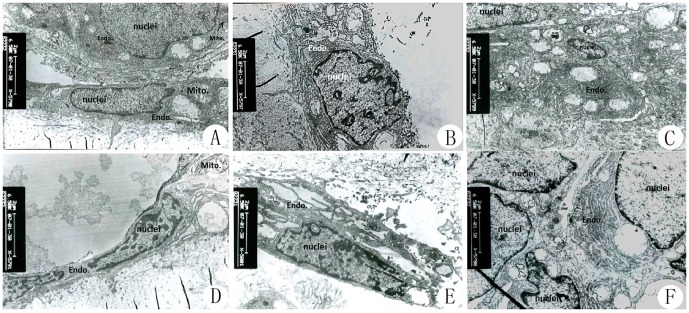
TEM (15000× magnification) of the femoral heads from each experimental group. Normal control group (A): Osteogenic cells were round or oval, large in size and rich in organelles. Model control group (D): Osteoblasts were flat with swollen mitochondria with degenerated vacuoles and relatively few rough endoplasmic reticula (rER) were present in the cytoplasm. Negative pressure group, week 4 (B) and week 8 (C): Osteoblasts were round or oval, the cells gradually increased in size and the number of rER increased in the cytoplasm and formed membranous tubes with attached ribosomes. In addition, there were numerous mitochondria. Nuclear staining was light and the structure was intact. Core decompression group, week 4 (E) and week 8 (F): ER and mitochondria numbers in the cytoplasm of the osteoblasts were increased, but increases were to a lesser extent compared with the negative pressure group at various time points.

Histological examinations revealed that at weeks 4 and 8 postoperatively, trabecular bone shape, empty lacunae and bone marrow hematopoietic cell and fat cell numbers were improved in the negative pressure group compared with the core decompression group (Figure 3). At week 8, there was no significant difference between the negative pressure and normal control groups. Immunohistochemistry revealed that VEGF and BMP-2 expression levels were higher in the negative pressure group compared with the core decompression group (Figure 4, 5). TEM revealed that at various time points, cellular organelles were further developed in the negative pressure group compared with the core decompression group (Figure 6). At week 8, cell structures did not differ between the negative pressure and normal control groups, and in both groups cell structure was normal with abundant organelles.

### Microvascular features

**Figure 7:**
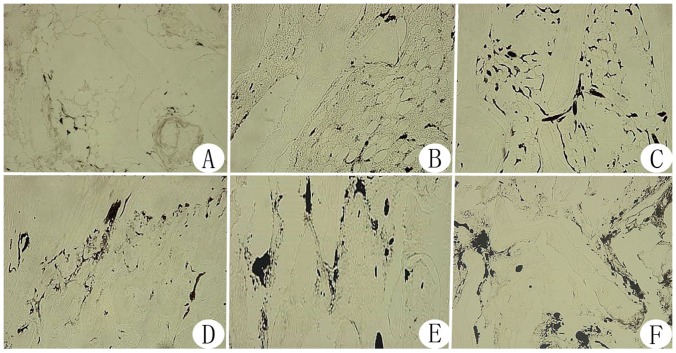
Femoral head microvascular ink staining images (100× magnification) from each experimental group. In the model control group, the small number of ink-stained blood vessels were thin and presented primarily in the bone marrow cavity of the femoral head (Figure A). In the normal control group, there were relatively more ink-stained blood vessels with a thick vascular lumen in the bone marrow of the femoral head (Figure D). Compared with the model control group, the core compression group had more ink-stained blood vessels with a thicker vascular lumen in the bone marrow of the femoral head at week 4 (B) and week 8 (C). However, there was no significant difference between week 4 and week 8. In the negative pressure group, the number of ink-stained blood vessels with a thicker vascular lumen in the bone marrow of the femoral head was increased significantly at week 4 (E) and week 8 (F). Compared with week 4, there was significant improvement at week 8, and the parameters at both time points were superior to those of the normal control group.

**Figure 8:**
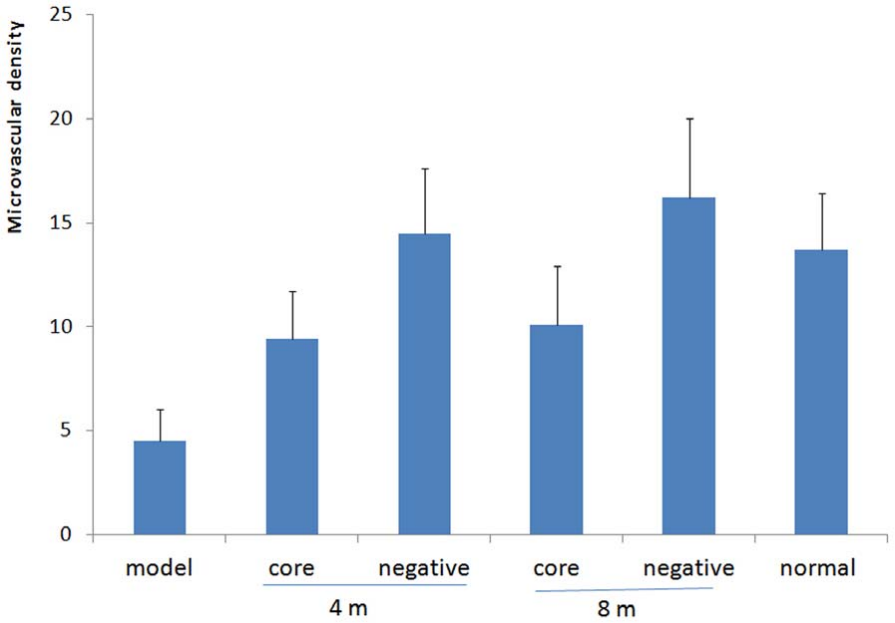
Comparison of femoral head microvascular density among experimental groups. Each experimental groups was significantly different compared with the model control group (*P*<0.01, n = 8). The negative pressure group and the core decompression group were significantly different at weeks 4 and 8 (*P*<0.01, n = 8).

Microvascular ink staining of the femoral head revealed that the number of ink-stained blood vessels in the bone marrow of the femoral head was increased, the vascular lumen was thickened and microvascular density increased in the negative pressure group compared with the core decompression group (Figure 7, 8).

### RT-PCR

**Figure 9:**
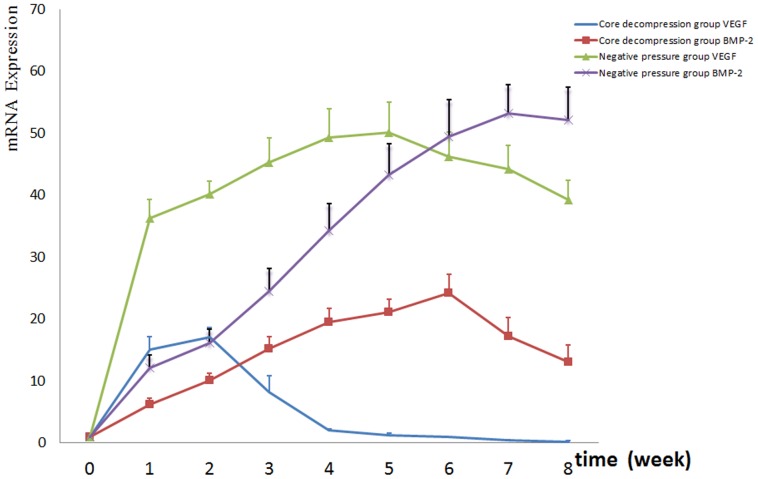
VEGF and BMP-2 mRNA levels over time in the core decompression and negative pressure groups. At weeks 1, 2, 3, 4, 5, 6, 7 and 8, VEGF and BMP-2 expression levels increased significantly and the peak expression levels lasted longer in the negative pressure group compared with the core decompression group (n = 6).

RT-PCR showed significant differences in the mRNA levels of both VEGF and BMP-2 among experimental groups (*P*<0.01). Compared with the core decompression group, mRNA levels of VEGF and BMP-2 were higher in the negative pressure group. In the core decompression group, VEGF expression peaked at weeks 1–2, with no difference compared to the model control group at week 4. However, VEGF expression was lower in the core decompression group compared with the normal control group. In the core decompression group, BMP-2 expression peaked at weeks 5–6 and began to decrease at weeks 7–8. However, BMP-2 expression in the core decompression group remained higher compared with the model control group. In the negative pressure group, VEGF expression peaked at weeks 4–5, and this prolonged peak expression level was three times that compared with the core decompression group. In the negative pressure group, BMP-2 expression peaked at weeks 6–7 and the peak expression level was more than two times that compared with the core decompression group (Figure 9).

## Discussion

Our results show that negative pressure has a therapeutic effect on FHN and is superior to core decompression. Previous studies have reported that a negative pressure of −50 kPa can induce human mesenchymal stem cells to differentiate into bone cells, promote osteoprotegerin (OPG) expression, and reduce OPG-ligand (OPGL) expression. In our study, we therefore applied 30 min episodes of 50 kPa twice daily. We were initially concerned that the placement of a negative pressure tube for six weeks might lead to infection. After surgery, we routinely used antibiotics, thereby allaying this concern. It was an important prerequisite for our study to establish a simple and easy-to-use animal model, consistent with clinical features. There are three principal methods for establishing an animal model of steroid-induced ischemic necrosis of the femoral head: the simple hormone model, the hormone-serum model, and the hormone-endotoxin model [12], [13]. We adopted the hormone-endotoxin model and successfully established the animal model by week 6. However, the degree of necrosis among the experimental rabbits varied and the experimental cycle was relatively long. This complicated our evaluation, despite increasing the number of animals.

Our histopathological studies showed that trabecular bone shape, empty lacunae, and bone marrow hematopoietic cell and fat cell numbers were improved in the negative pressure group compared with the core decompression group. Furthermore, the core decompression group yielded enhanced results compared with the model control group but less favorable results compared with the normal control group. At week 8, there was no difference between the negative pressure and normal control groups, suggesting that negative pressure exerted a therapeutic effect on FHN. Cellular microstructural and histology studies revealed similar results, with the negative pressure group containing enhanced numbers of organelles and the highest metabolism. Microvascular studies of the femoral head revealed that negative pressure can improve the microvascular conditions needed for repairing bone tissue. In our study, biomechanical indicators were not investigated, which we believe is an important aspect to investigate in a future study.

When compared with the core decompression group, the negative pressure group had higher VEGF and BMP-2 expression levels. VEGF mRNA expression in the core decompression group peaked at weeks 1–2, was comparable to the model control group at week 4, but was lower compared with the normal control group. BMP-2 mRNA expression in the core decompression group peaked at weeks 5–6 and began to decrease at weeks 7–8. BMP-2 expression lagged behind expression of VEGF. The short-term effect of core decompression on FHN may be closely related to factors that lead to FHN. Although core decompression can appear to reduce the pressure and improve microcirculation in the bone marrow and temporarily slow the process of bone necrosis, other factors remain. These include osteonecrosis-causing pathological changes that underlie the increased pressure in the bone marrow, altered vascular regeneration in the necrotic femoral head, decreased repair and regeneration capacities of bone cells and increased osteoclast activity. Core decompression alone does not prevent this series of pathological changes [14]–[16]. The level of VEGF mRNA in the negative pressure group was three times that of the core decompression group, and this elevated VEGF expression level was longer-lasting. The amplitude of the peak in BMP-2 expression was more than two times the level in the core decompression group. Combined with histology results, there are many explanations for the superior results observed in the negative pressure group. For example, the application of continued intermittent negative pressure achieved simple, controlled, repeated decompression *in vivo*, with a significantly superior decompressive effect than core decompression alone. Moreover, the contributing effect of applying negative pressure on tissue revascularization led to improved microcirculation in the femoral head lesion, increased the formation of new bone, accelerated the process of creeping substitution of bone tissue, improved the environment in the bone marrow cavity, decreased intraosseous pressure and relieved circulatory stasis. As a result of stretch-induced stress, VEGF and BMP-2 expression gradually increased and their angiogenic and osteogenic effects were intertwined. The combined influence of these mechanical and subsequent biological effects accelerated the process of repairing early FHN.

Achieving the ideal treatment for FHN is dependent on reaching an early diagnosis. Prior to X-ray assessment, certain measures are needed to prevent femoral head collapse and to preserve joint function. Early treatment methods are focused primarily on non-surgical options and palliative surgery and can include avoiding weight-bearing activities, the use of pharmaceuticals, electrical stimulation, hyperbaric oxygen, interventional therapy, core decompression and vascularized bone grafts. However, all of these methods fail to effectively slow the progress of FHN, and their efficacy is generally unsatisfactory [17]. The eventual collapse of the femoral head in many patients leads to both osteoarthritis of the hip joint and joint dysfunction.

In our study, we used the endotoxin-hormone FHN model and negative pressure to treat early FHN in rabbits. We evaluated the effect of applying negative pressure on FHN and analyzed the expression levels and distribution patterns of BMP-2 and VEGF using histomorphology, microvascular ink staining, immunohistochemical staining and RT-PCR. Our results demonstrate that applying negative pressure can significantly increase expression levels of BMP-2 and VEGF in the rabbit femoral head, with a superior therapeutic effect being achieved on FHN than was accomplished through core decompression. Our study shows that applying negative pressure has potential for treating early FHN. Further clinically relevant studies are needed to evaluate the efficacy, safety and feasibility of using the negative pressure membrane technique and to lay the foundation for its clinical application.
